# Similar does not mean the same: ERP correlates of mental and physical experiencer verb processing in Malayalam complex constructions

**DOI:** 10.3389/fnhum.2025.1632844

**Published:** 2025-08-11

**Authors:** S. Shalu, R. Muralikrishnan, Kamal Kumar Choudhary

**Affiliations:** ^1^Department of Humanities and Social Sciences, Indian Institute of Technology Ropar, Rupnagar, India; ^2^Max Planck Institute for Empirical Aesthetics, Frankfurt, Germany

**Keywords:** mental experiencer verb, physical experiencer verb, N400, LAN, Malayalam

## Abstract

This study examined the neurophysiological correlates of processing mental experiencer verbs and physical experiencer verbs in Malayalam complex constructions, where the subject argument assumed the role of the experiencer. Event-related brain potentials (ERPs) were recorded as 28 first-language speakers of Malayalam read intransitive sentences with the two types of experiencer verbs. The subject case either matched (acceptable) or violated (unacceptable) the requirements of the verb in the critical stimuli. Both mental and physical experiencer verbs engendered negative effects in the 400–600-ms time window when the subject case did not match the verb’s requirements. Additionally, mental experiencer verbs evoked a left anterior negativity LAN-like effect in the same time window, regardless of grammaticality. Thus, even though both kinds of experiencer verbs are processed qualitatively similarly, inherent differences between mental and physical experiencer verbs in Malayalam persist and are discernible.

## 1 Introduction

Interest in the processing of different verb types and their cross-linguistic applicability has been growing over the past few decades. While early studies focused on broad classifications of verbs, more recent research has explored increasingly detailed subcategories, highlighting the nuanced distinctions in verb processing. One area of investigation has been the processing of subcategories of concrete verbs (Bedny et al., 2008; Kemmerer et al., 2008; Thomas et al., 2012; Dalla Volta et al., 2014; Repetto et al., 2015) and their comparison with abstract verbs (Binder et al., 2005; Goldberg et al., 2007; Hoffman et al., 2015; Hoffman et al., 2010; Perani et al., 1999; Sabsevitz et al., 2005). A few studies have examined the processing of different types of abstract verbs (Rodríguez-Ferreiro et al., 2011; Muraki et al., 2020; Dreyer et al., 2015; Dreyer and Pulvermüller, 2018). A notable contribution in this regard is that of Muraki et al. (2020), who argue that treating abstract verbs homogeneously limits our understanding of their underlying representation. In line with this, we consider verbs that do not refer to tangible motor actions as abstract verbs and assume abstractness to be a matter of degree.

The majority of existing ERP studies on abstract verbs have been conducted within the framework of embodied cognition theories. A central finding from this line of research is that different types of abstract verbs are represented differently in the brain (Muraki et al., 2020; Rodríguez-Ferreiro et al., 2011; Dreyer et al., 2015; Dreyer and Pulvermüller, 2018). However, there is limited research on processing different categories of abstract verbs based on their argument structure and realization. One of the verb categories examined in this regard is experiencer verbs and their major subcategories, such as subject experiencer (SE) verbs and object experiencer (OE) verbs. Several behavioral studies have examined the processing differences between SE and OE, as well as how these verbs differ from action verbs, revealing that SE are easier to process than OE because of their less complicated alignment encoding grammatical relations and thematic roles (Cupples, 2002; Kretzschmar et al., 2011; Gattei et al., 2015; Gattei et al., 2017; Gattei et al., 2022; Mateu, 2022; Wilson and Dillon, 2022). To our knowledge, no study has yet examined the processing of experiencer verbs within the SE category, except for Shalu et al. (2025), who investigated mental experiencer (ME) verbs (indicating mental experiences like happiness, sadness, etc.) and physical experiencer (PE) verbs (indicating physical experiences like hunger, thirst, cold, etc.) in Malayalam—a Dravidian language that clearly distinguishes between these verbs in its syntax.

**Table:** 

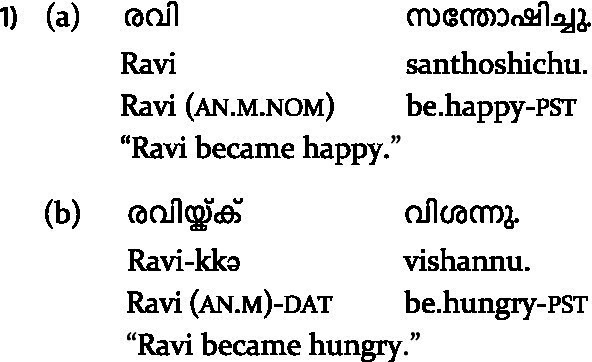

In simple experiencer constructions, ME requires a nominative subject (1a), while PE requires a dative subject (1b) in Malayalam. Shalu et al. (2025) crossed subject case and verb type in their design and reported N400s for both ME and PE when the subject was anomalous. However, they also found differences in the peak latency of the effect between ME and PE, as well as an influence of sentence-final acceptability on the ERPs for the non-anomalous conditions, suggesting subtle but robust differences in processing ME vs. PE.

However, a key limitation of their design is that it does not allow for disentangling the effect of the verb type *per se* from the effect of differing expectations arising from the interaction between the sentence-initial nominative vs. dative subject and the verb type. This is because the subject case (say, nominative) that constituted a violation for PE was, by contrast, the non-anomalous subject case for ME, and vice versa. A potential way to address the issue would be to examine ME and PE in complex predicates, since both verb types require a dative subject in these constructions (2a–b).

**Table:** 

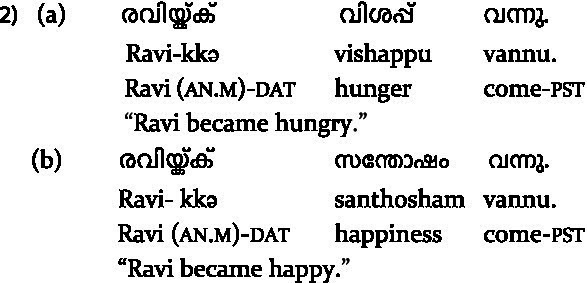

### 1.1 The present study

We employed complex intransitive constructions in the present study, formed using an event nominal (physical: “*pani*” fever, or mental: “*santhosham*” happiness) that provides most of the meaning and activates its argument structure, followed by a semantically underspecified light verb (“*vannu*’ come). We employed a 2 × 2 factorial design, similar to that of Shalu et al. (2025), manipulating the subject case (nominative or dative) and predicate type (ME or PE), keeping the light verb identical across conditions. Since the sentence-initial subject was uniformly dative in all non-anomalous conditions and nominative in all violation conditions, any ERP differences at the verb for ME violations vs. PE violations would be directly attributable to a processing difference between the two verb types.

Our hypotheses were as follows. If ME and PE in Malayalam complex constructions are processed similarly, then qualitatively similar ERP correlates should be expected for both verb types.

- This would be in line with the theoretical literature on experiencer verbs since the argument structure is identical for both ME and PE (Subject_DAT-EXP_-Verb_MENT/PHY_).
- Following Shalu et al. (2025), we expected an N400 effect for both the violation conditions compared to their respective correct counterparts as a marker of the violation of an interpretively relevant linguistic rule.

However, since a large part of the predicative meaning originates from the event nominal within the complex construction (Butt, 2010), and the nominals in the study represent two very different types of experiences, namely, mental vs. physical (Jayaseelan, 2004), we should observe qualitative neurophysiological differences between the two verb types.

- While the exact nature of this difference remains to be seen, observing such a difference would add to the claim from Shalu et al. (2025) that the two types of experiencers show inherent processing differences despite their overall processing similarity in Malayalam.
- Further, if the differences in peak latency for ME vs. PE in Shalu et al. (2025) were due to inherent distinctions between these verb types *per se*, we should expect to see similar differences in the present study. However, if these differences were stemming from nominative vs. dative subject case interacting with verb type, no such difference should ensue.

## 2 Method

### 2.1 Participants

Twenty-eight first-language speakers of Malayalam (mean age = 27.8; 11 women and 17 men) participated in the experiment in exchange for payment. They had learned Malayalam before the age of six and studied the language as an academic subject in school until 10th grade. All participants were dominantly right-handed, as determined by an adapted version of the Edinburgh Handedness Inventory (Oldfield, 1971) in Malayalam. Data from seven participants were excluded from analysis due to excessive artifacts in their data epochs as determined by an amplitude threshold of ±100 μV (further details are provided in the Supplementary material).

### 2.2 Materials

We employed critical stimuli in 4 conditions (Box 1), with 36 sentences in each critical condition, resulting in a total of 144 critical sentences. These were interspersed with fillers and pseudorandomly presented. Further details are provided in the Supplementary material.

**Box 1:** Experiment Stimuli of the present study.

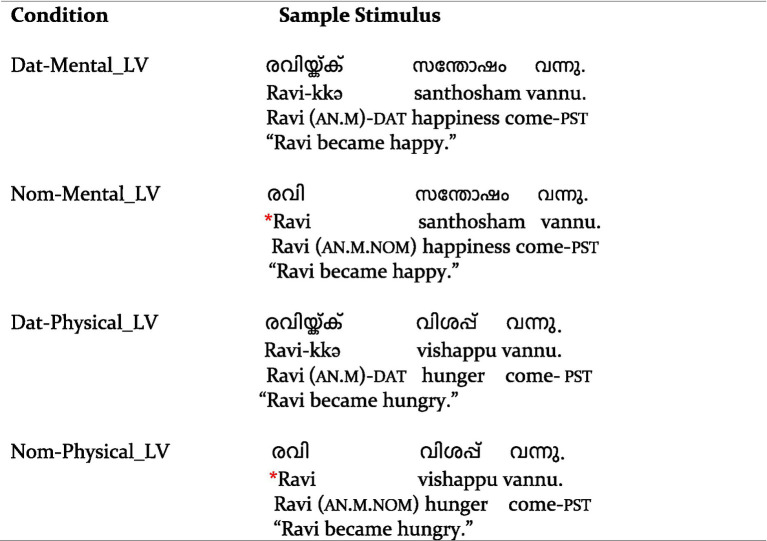

Experiencer light verbs are indicated as Mental_LV (mental experiencer) and Physical_LV (physical experiencer), and case markers are indicated as Nom (nominative) and Dat (dative). Dat-Mental_LV and Dat-Physical_LV are grammatically correct sentences, and Nom-Mental_LV and Nom-Physical_LV are ungrammatical sentences.

### 2.3 Procedure

Participants were seated in a dimly lit, sound-attenuated room during the experiment, which included a brief practice session. Stimuli were presented using E-Prime 2.0 (Psychology Software Tools, Pittsburgh, PA, United States) in a rapid serial visual presentation. Participants performed an acceptability judgment and a probe task after each trial. Further details are provided in the Supplementary material.

### 2.4 EEG recording, pre-processing, and statistical processing

Scalp activity was recorded using 32 Ag/AgCl electrodes fixed to the scalp using Hydrocel Geodesic Sensor Net 32, with Cz as the online reference. EEG data were pre-processed using EEGLAB (version 14; Delorme and Makeig, 2004, sccn.ucsd.edu) in MATLAB (version R2023b; The MathWorks, Inc.). Data epochs were extracted at the critical position (verb; −200 to 1,200 ms) from the continuous recordings for each participant and analyzed using the eeguana package (version 0.1.11.9001; Nicenboim, 2018) in R (version 4.4.3; R Core Team, 2024).

#### 2.4.1 ERP data analysis

The mean amplitudes in the time window of interest were statistically analyzed using the single-trial EEG epochs at the verb for each critical condition by fitting linear mixed effects models in R (version 4.4.3, R Core Team 2024) using the lme4 package (Bates et al., 2015). The statistical models included the fixed factors Case (Nominative vs. Dative) and Verb type (Mental experiencer vs. Physical experiencer), as well as the topographical factor Regions of Interest (ROI). We included the mean amplitude from the 200-ms pre-stimulus period (−200 to 0 ms) as a (scaled and centered) covariate in the model for each data epoch (Alday, 2019). Categorical fixed factors used sum contrasts (scaled sum contrasts for two-level factors) so that the coefficients represent deviations from the grand mean (Schad et al., 2020). The ROIs were defined by clustering topographically adjacent electrodes in 6 lateral and 2 midline regions. The lateral ROIs were as follows: left-frontal, which included electrodes E3 and E11 (which, in the 10–20 electrode system, would have been equivalent to F3 and F7); left-central, which included electrodes E5 and E13 (C3 and T7); left-parietal, which included electrodes E7 and E15 (P3 and P7); right-frontal, which included electrodes E4 and E12 (F4 and F8); right-central, which included electrodes E6 and E14 (C4 and T8); and right-parietal, which included electrodes E8 and E16 (P4 and P8). Mid-fronto-central, which included E17 and E28 (Fz and ~FCz), and mid-parieto-occipital, which included E19, E20, E9, and E10 (Pz, Oz, O1, and O2), were the midline ROIs. Further details are provided in the Supplementary material.

## 3 Results

### 3.1 Behavioral data

**Figure 1:**
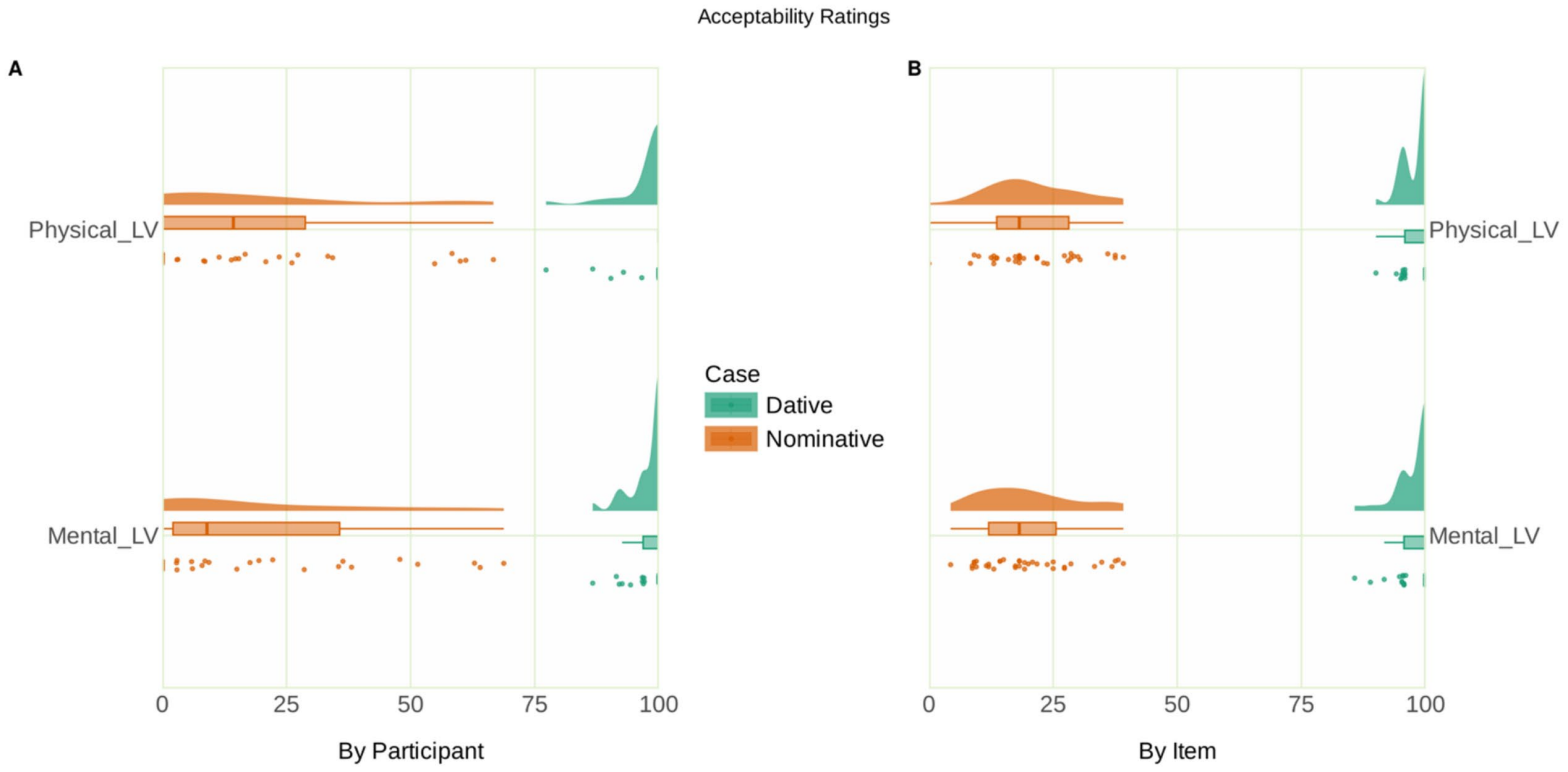
Raincloud plot of the acceptability ratings. **(A)** The by-participant variability of acceptability ratings, with the individual data points representing the mean by-participant acceptability of each case and verb type combination. **(B)** The by-item variability of acceptability ratings, with the individual data points representing the mean by-item acceptability of each case and verb type combination.

The mean acceptability ratings and probe detection accuracy for the critical conditions were calculated for the trials included in the ERP analysis. As Table 1 shows, the non-anomalous conditions were rated as highly acceptable (>98%; ceiling), whereas the acceptability for the violation conditions was very low across the board (<21%). The probe detection accuracy reached a ceiling for all the conditions. Figure 1 shows raincloud plots (Allen et al., 2021) of the behavioral acceptability judgments.

**Table 1:** Mean acceptability ratings and probe detection accuracy.

Case	Verb type	Mean acceptability % (SD)	Mean accuracy % (SD)
Nom	Mental_LV	19.8 (39.9)	96.7 (17.9)
Dat	Mental_LV	98.0 (14.0)	97.6 (15.2)
Nom	Physical_LV	20.8 (40.6)	97.1 (16.9)
Dat	Physical_LV	98.4 (12.6)	97.6 (15.2)

Experiencer light verbs are indicated as Mental_LV (mental experiencer) and Physical_LV (physical experiencer), and case markers are indicated as Nom (nominative) and Dat (dative). Dat-Mental_LV and Dat-Physical_LV are grammatically correct sentences, and Nom-Mental_LV and Nom-Physical_LV are ungrammatical sentences.

The behavioral acceptability and accuracy were analyzed by fitting generalized linear mixed models using the lme4 package in R. Categorical fixed factors used scaled sum contrasts (effect coding). In the analysis of acceptability data, the statistical model included the fixed factors Case (Nominative vs. Dative) and Verb type (Mental experiencer vs. Physical experiencer), with random intercepts for participants and items and by-participant random slopes for the effects of Case, Verb type, and their interaction term. Type II Wald chi-squared tests of the fitted model (AIC = 1571.012) of the acceptability data showed a main effect of Case [*χ*^2^(1) = 109.66, *p* < 0.001, *s* = 82.83] and the interaction of Verb × Case [*χ*^2^(1) = 7.49, *p* = 0.006, *s* = 7.33]. Estimated marginal means on the response scale were computed on the model using the emmeans package (Lenth Russell, 2021) to resolve this interaction, which showed that, for both verb types, the estimates for the conditions with a dative subject were higher compared to those for conditions with a nominative subject. Pairwise contrasts of these estimates within each verb type revealed a simple effect of Case for both mental experiencer light verbs [estimate = 0.878, standard error (SE) = 0.038, *p* < 0.001, *s* = 373.79] and physical experiencer light verbs [estimate = 0.873, SE = 0.040, *p* < 0.001, *s* = 346.06]. These findings reflect the substantial difference in acceptability between conditions with dative subjects, which engendered very high acceptability, vs. conditions with nominative subjects, which were rated as hardly acceptable.

In the analysis of probe detection accuracy, models with an interaction term of the fixed factors Verb and Case, as well as those with random slopes, were singular. The model with Verb and Case as fixed factors with by-participant and by-item random intercepts (AIC = 816.1534) did not detect any effect involving Verb or Case (*s* < 2.5), reflecting the highly similar (ceiling) accuracy of probe detection across conditions.

### 3.2 ERP data

**Figure 2:**
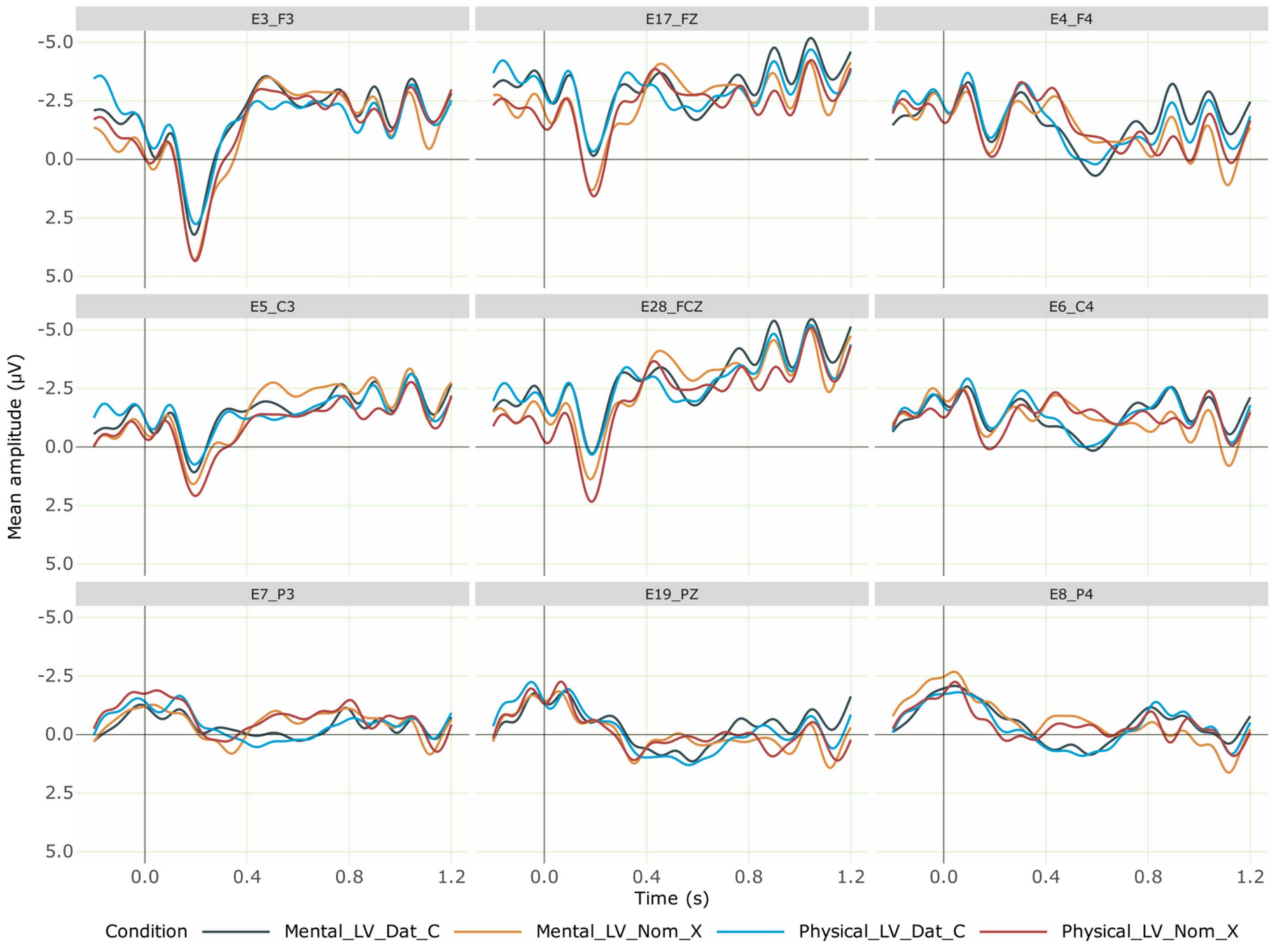
Grand averaged ERPs at the verb for the critical conditions across 28 participants. Negativity is plotted upwards; the time axis runs from −0.2 s to 1.2 s (i.e., −200 ms to 1,200 ms) with 0 being the onset of the critical verb. The dark blue line represents the correct mental experiencer light verb (with a dative subject). In contrast, the orange line shows the incorrect mental experiencer light verb (with a nominative subject), which elicited a centroparietal negativity effect. The light blue line indicates the correct physical experiencer verb (with a dative subject) and the red line represents the incorrect physical experiencer verb (with a nominative subject), which elicited a centroparietal negativity effect. Regardless of grammaticality, conditions with mental experiencer light verbs elicited a further negativity effect in the left anterior region compared to physical experiencer light verbs.

The ERPs at the verb are shown in Figure 2. Visual inspection of the ERP data indicated a negativity effect in the violation conditions relative to the non-anomalous ones. Based on both the ERP components identified in previous research (Shalu et al., 2025) and visual inspection, we selected the 400–600-ms time window for analysis. The single-trial ERP mean amplitudes extracted in the analysis time window from a total of 3,238 data epochs entered the analysis. The raw data collected during the experiment, the pre-processing pipeline used, and the pre-processed data are available in the online data repository.

We computed a linear mixed-effects model with the fixed factors Verb type, Case of the subject noun, ROI, the −200 to 0-ms pre-stimulus baseline mean amplitude as a covariate (scaled and centered), along with by-participant and by-item random intercepts. The analysis code and full model outputs are available as R notebooks in the online analysis repository. Type II Wald Chi-squared tests on this model (AIC = 434325.1) showed a main effect of Case [*χ*^2^(1) = 6.84, *p* = 0.008, *s* = 6.81] and interaction effects of ROI × Verb [*χ*^2^(1) = 14.79, *p* = 0.03, *s* = 4.69] and ROI × Case [χ^2^(1) = 14.82, *p* = 0.03, *s* = 4.70]. Estimated marginal means on the response scale were computed using the emmeans package (Lenth, 2021) in the model to resolve these interactions. The pairwise contrasts of estimates for Case within each level of ROI revealed simple effects of Case in the right-frontal (estimate = 1.245, SE = 0.364, *p* < 0.001, *s* = 10.60), right-central (estimate = 1.067, SE = 0.364, *p* = 0.003, *s* = 8.19), right-parietal (estimate = 1.012, SE = 0.364, *p* = 0.005, *s* = 7.50), mid-fronto-central (estimate = 0.947, SE = 0.365, *p* = 0.009, *s* = 6.71) and mid-parieto-occipital (estimate = 0.566, SE = 0.319, *p* = 0.07, *s* = 3.71) regions. The estimates for nominative subjects were more negative than those for dative subjects in these regions. The pairwise contrasts of estimates for Verb type within each level of ROI revealed a simple effect of Verb type in the left-frontal region (estimate = −0.839, SE = 0.364, *p* = 0.02, *s* = 5.54). The estimate for mental experiencer verbs was more negative than that for physical experiencer verbs in this region. A more complex model in which the by-participant random slopes for Case, Verb type, and their interaction term were included in the random effects specification^1^ showed that this pattern of results remained largely intact despite numerical differences. In sum, both mental and physical experiencer verbs elicited a negativity effect when the subject was in the nominative case as opposed to the dative case. Further, a general verb-type effect ensued for mental experiencer verbs, which elicited a negativity effect in the left-anterior region compared to physical experiencer verbs, regardless of grammaticality.

## 4 Discussion

We have presented an ERP experiment on Malayalam, which aimed to examine whether the complex ME and PE are processed qualitatively similarly. The behavioral task demonstrated a clear link between grammaticality and acceptability, with violation conditions leading to a drastically low acceptability compared to grammatical sentences. The ERP results showed a negativity effect in the 400–600-ms time window, which can be plausibly interpreted as an instance of an N400 effect for violations involving both verb types. More interestingly, however, there was an unexpected left-lateralized anterior negativity effect in the same time window for ME regardless of grammaticality; that is, the effect was a general verb type effect observed for both anomalous and non-anomalous ME in comparison to PE. Although reminiscent of the classic focal left anterior negativity (LAN), since our critical stimuli did not constitute a morphosyntactic violation of the type typically associated with that effect, we tentatively call the effect we found “LAN-like.” In addition to this contrast to findings from Shalu et al. (2025), the current study did not observe any peak latency differences in the negativity effect between ME and PE. This can be attributed to the fact that both conditions in the present study begin with a nominative argument, thus generating identical expectations about possible continuations. In both cases, these expectations are violated similarly when the parser encounters the ME or PE verb. This finding supports the notion that the latency differences reported by Shalu et al. (2025) may have been attributable to the violation of expectations based on the nominative vs. dative subject arguments, rather than reflecting an inherent distinction between ME and PE *per se*.

### 4.1 N400 effect

The N400 effect found for both ME and PE is perhaps unsurprising because both verb types have an identical argument structure and realization (Belletti and Rizzi, 1988; Grimshaw, 1990; Levin and Hovav, 2005). This result is consistent with findings from Shalu et al. (2025). As in their study, we interpret the negative effects that we found in the present study as a consequence of a violation of interpretively relevant rules, which aligns with Choudhary et al. (2009) and Nieuwland et al. (2013).

In Malayalam, a clear morphosyntactic constraint governs the case marking of the subject argument in ME and PE in complex constructions. Regardless of whether the experience meant by the experiencer verb is mental or physical, it is expressed as though it is moving from an abstract space to the subject argument, which serves as the experiencer-goal of the abstract movement. In this respect, the dative subject experiencer is similar to other dative arguments that are the targets/goals of some movement (see sentences 5–7). Consequently, the subject is in the dative case, which is the general obligatory marker for targets of movement in Malayalam (Nizar, 2010; Mohanan, 1990; Krishnan, 2020; Jayaseelan, 2004). The violation of this interpretively relevant rule in the present study led to an N400 effect for both verb types, regardless of the experience conveyed by the verb.

**Table:** 

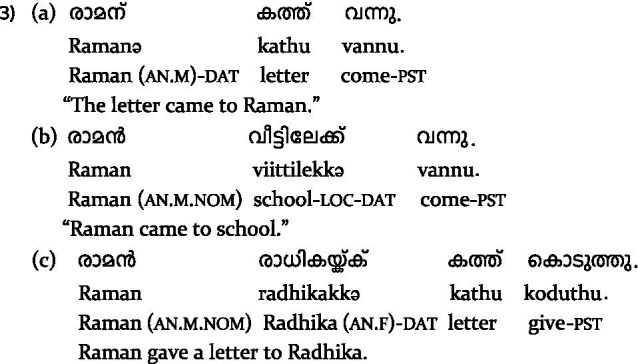

### 4.2 LAN-like effect

The classic focal LAN effect is typically elicited by anomalous inflectional morphology (Gunter et al., 2000) and is generally understood as an indicator of the detection of morphosyntactic inconsistencies (Münte et al., 1998; Friederici, 2002; see Molinaro et al., 2011 for a review). It is observed in the same time window as the N400, with often the only difference between the two effects being the topography. Although the effect we found for ME is in the left-anterior region and within the same time window as the N400 effect, there are at least two points that speak against interpreting this LAN-like effect as an instance of the classic focal LAN. First, as already mentioned, our critical stimuli did not constitute a morphosyntactic violation of the type typically associated with the LAN. Second, and more importantly, the LAN-like effect is a general verb-type effect for ME, regardless of anomaly. That is, the same effect is elicited by both anomalous and non-anomalous sentences of the ME type.

Instead, a finding that would be more pertinent for interpreting this effect stems from previous imaging studies on abstract verbs, which have shown that left-lateralized brain regions are activated when processing these verbs (Rodríguez-Ferreiro et al., 2011; Binder et al., 2005; Sabsevitz et al., 2005; Noppeney and Price, 2004; Perani et al., 1999). In line with this, it is plausible that the LAN-like effect observed in our study for ME could be explained by the degree of abstractness and non-bodily involvement associated with these verbs compared to PE (Borghi, 2020). ME refers to internal states, often linked to emotions, thoughts, etc., and other intangible aspects that cannot be directly observed. By contrast, PE describes physical sensations or states. Compared to ME, which does not directly engage with the body, PE is closely linked to the physical body and the tangible sensations it can perceive and feel. That is, even though PE is abstract, they have some observable effects in the body that make it more perceivable compared to relatively more abstract mental experiences. For instance, an individual suffering from fever may have a flushed face and sweating, an individual who is experiencing hunger may have a growling sound from the stomach, etc. Thus, the LAN-like effect observed for ME can be plausibly attributed to their more abstract nature compared to PE. This interpretation is necessarily tentative, and further neurophysiological research on the finer distinctions of abstract verbs would be needed to confirm this.

Alternatively, the LAN-like effect for ME in comparison to PE could plausibly be explained in terms of the ± control/agency associated with the respective verb types (Schlesinger, 1992; Mohanan, 1990; Jayaseelan, 2004). ME refers to a state where the experiencer has volitional control/agency over their mental state, allowing them to change or influence it without taking physical action. For instance, someone can willfully suppress sadness to maintain composure. In contrast, PE describes a state where the experiencer has little control/agency over the situation and must take tangible actions to change it. For example, a person experiencing freezing cold must actively do something to alleviate the discomfort, such as wearing warm clothes or turning on the heater. In light of this, it is plausible to consider the LAN-like effect observed in our study as a marker of the control/agency difference between ME and PE. However, this interpretation also remains speculative, and more studies comparing agentive verbs with non-agentive verbs would be needed to gain further insights in this regard.

Verb types differ in terms of their internal semantic structure (Davidson, 1971; Dowty, 1979; Jackendoff, 1991; Levin and Hovav, 2005), and several processing studies have attributed the differences observed between verb types to this aspect. However, a limitation of many of these studies is that it is very difficult to isolate syntactic differences from semantic differences (Brennan, 2015). Our stimuli did not have this issue because all critical sentences in our study were syntactically identical and differed only with respect to the type of experiencer verb. Therefore, the LAN-like effect that we found for ME regardless of grammaticality in comparison to PE appears to show inherent semantic differences between these two verb types, including but not limited to their degree of abstractness and agency.

In conclusion, the present study explored the processing of mental and physical experiencer verbs in Malayalam complex experiencer constructions. Results revealed a negativity effect for subject case violations involving mental as well as physical experiencer verbs, suggesting that they are processed qualitatively similarly. However, we also found a LAN-like impact for mental experiencer verbs, regardless of grammaticality, compared to physical experiencer verbs. In sum, although both mental and physical experiencer verbs are processed qualitatively similarly, our data nevertheless provide converging evidence to conclude that effects of inherent differences between mental and physical experiencer verbs in Malayalam are still discernible even in syntactically identical constructions involving the two verb types.

## Data Availability

The statistical analysis code and full model outputs of all the analyses reported are available as R notebooks here: https://zenodo.org/records/15478572.

## Footnotes

^1^Since the more complex model was nearly equivalent to the simpler model in terms of fit, predictive accuracy, and the amount of variance explained, we retain the simpler model for further interpretation. The full model output for the complex model is provided for reference in the online analysis repository.
