# Supplementary material for: Similar does not mean the same: ERP correlates of mental and physical experiencer verb processing in Malayalam complex constructions
Source: Front Hum Neurosci. 2025 Aug 11;19:1632844. doi: 10.3389/fnhum.2025.1632844 (PMC12376169; doi:10.3389/fnhum.2025.1632844)
Supplement: Supplementary file 1 [file Supplementary_file_1.docx]

Supplementary Material

**Similar does not mean same: ERP correlates of processing mental and physical experiencer verbs in Malayalam complex constructions**

**S. Shalu^1^, R. Muralikrishnan^2*^ & Kamal Kumar Choudhary^1^**

*^1^Department of Humanities and Social Sciences, Indian Institute of Technology Ropar, Rupnagar, India.*

*^2^Max Planck Institute for Empirical Aesthetics, Frankfurt, Germany.*

***Correspondence**

R. Muralikrishnan,

Max Planck Institute for Empirical Aesthetics,

Grüneburgweg 14, 60322 Frankfurt am Main, Germany.

Phone: +49 69 8300 479 615
E-mail: r.muralikrishnan@aesthetics.mpg.de

**A1. Participants**

A total of 28 first-language speakers of Malayalam (mean age = 27.8; age range = 18–40; 11 female and 17 male), primarily students and staff at the Indian Institute of Technology Ropar, residing in Ropar, India, participated in the experiment in exchange for payment. Prior to the experiment, participants were informed about the study, and written consent was obtained for the use of their data for academic purposes. The research protocol was approved by the Ethics Committee (Human) of the Indian Institute of Technology Ropar. All participants were right-handed, as determined by an adapted version of the Edinburgh Handedness Inventory (Oldfield, 1971) in Malayalam. They had normal or corrected-to-normal vision and were free from known neurological disorders at the time of the experiment. All participants were native Malayalam speakers, having acquired the language before the age of six. Additionally, most spoke other languages. Data from 7 participants were excluded from analysis due to excessive artifacts.

**A2. Materials**

In the critical conditions, we employed 9 mental experiencer (ME) complex predicates and 9 physical experiencer (PE) complex predicates. Each verb was repeated 4 times with 4 different nouns (2 masculine and 2 feminine nouns) which resulted in 36 sets of sentences with 144 critical sentences as in Table 2 in the main article. The light verb was identical across all sentences (“vannu-come”). The sentence-initial noun was either nominative (null case marking) or dative marked. Since Malayalam dative case marker has two allomorphs (“-kkə” or the “-ə” markers^[[1]](#footnote-1)^), we used an equal number of nouns requiring either the “-kkə” or the “-ə” marker (18 each). In these 144 critical sentences, 72 sentences were grammatically correct (dative good), and 72 sentences were grammatically incorrect (nominative bad). Additionally, we used 288 fillers, which were constructed to introduce diverse structures in the stimuli so as to avoid strategic responses from participants. They also served to counterbalance the number of grammatical and ungrammatical sentences with different subject types (72 good nominative sentences, 72 bad nominative sentences, 72 bad dative sentences and 72 good dative sentences).  The fillers were interspersed with the critical stimuli and pseudorandomized for presentation during the experiment.

**A3. Procedure**

The experiment began with a practice session, followed by the actual experiment, with all activities, including electrode preparation and stimulus presentation, taking approximately 3 hours. Participants were first briefed about the procedure and the tasks they would perform, and were provided with a printed instruction sheet. However, the specific research question was not disclosed to ensure unbiased data collection. Participants then filled out a consent form to provide informed consent for their participation, along with a Malayalam version of the Edinburgh Handedness Inventory to assess their handedness. Next, their head measurements were taken, and the Hydrocel GSN net was applied to their scalp. They were seated comfortably in a soundproof chamber, positioned 1 meter from a 20” LCD monitor where the stimuli were displayed. Stimuli presentation was managed using E-prime 2.0 (Psychology Software Tools, Pittsburgh, PA).

The structure of a trial in the experiment was as follows: sentences were presented visually, chunk-by-chunk, at the centre of the monitor screen. At the start of each trial, participants saw a "+" fixation sign at the centre of the screen for 1000 ms. This was followed by a blank screen for 100 ms. Each chunk was presented for 650 ms, followed by an interstimulus interval (ISI) of 100 ms (Muralikrishnan & Idrissi, 2021; Demiral et al., 2008; Bornkessel-Schlesewsky et al., 2020). After the fixation point, a noun phrase (NP) appeared on the screen, followed by a verb (critical position for the study). After this, participants performed two tasks. Since a violation paradigm was used in the study, they first completed an acceptability judgment task. A “???” sign appeared on the screen to signal the acceptability judgment task. They had to press the green button on the response pad if they found the preceding sentence acceptable, or the red button if they found it unacceptable. Additionally, to ensure the participants were paying attention to the sentence, they also completed a probe task. After finishing the acceptability judgment task or once 1500 ms had passed, a probe word appeared on the screen for 2000 ms. Participants were required to press the green button if the probe word was present in the preceding sentence or the red button if it was not. For instance, an experimental sentence like “menuvinu (thanuppu vannu)” (“Meenu became cold”) might be followed by a probe word “meenuvinu” or an incorrect probe word like “kappal” (“ship”). Half of the probe words required a ‘yes’ response, while the other half required a ‘no’ response.

The experiment was conducted in two versions: in one version, the correct response was mapped to the left key and the incorrect response to the right key, while in the other version, this mapping was reversed. Participants were instructed to avoid blinking during the stimulus presentation but were allowed to blink while performing the tasks. Prior to the actual experiment, they underwent a practice session to familiarize themselves with the trial structure, although none of the real experimental stimuli were included in the practice session. The experimental session was divided into 9 blocks, each containing 48 sentences, with short breaks at the end of each block. At the conclusion of the experiment, participants were asked to complete a questionnaire about their experience.

**A.3.1. EEG recording, Pre-processing and Statistical processing**

Scalp activity was recorded by means of 32 Ag/AgCI electrode fixed at the scalp by means of Hydrocel geodesic Sensor Net 32 channel (reference). Cz served as the online reference. To monitor EOG (Electro-oculogram) data, electrodes were placed as follows: for horizontal eye movements, electrodes were positioned at the outer canthus of each eye, and for vertical eye movements, electrodes were placed below the eyes. The interelectrode impedance was maintained below 50 kΩ (with an amplifier input impedance greater than 1 GOhm) (Ferree et al., 2001). All EEG and EOG data were amplified using a Net Amps 400 Amplifier and recorded at a sampling rate of 500 Hz.

The EEGLAB toolbox (Version 14; Delorme & Makeig, 2004, sccn.ucsd.edu) in MATLAB (Version R2023b; The MathWorks, Inc.) was used for the pre-processing of EEG data. The data was down-sampled to 250 Hz and filtered with a 0.3–20 Hz band-pass filter to eliminate slow drifts. These settings encompass the typical 0.5–5 Hz range for language-related ERP activity (Delorme, 2023; Roehm et al., 2002) and have been widely used in cross-linguistic ERP studies on language processing. Channels on the outer extent of the face and head were eliminated after this data was offline-referenced to the mean of the two mastoids. A copy of this original data that had been high-pass filtered at 1 Hz was subjected to an Independent Component Analysis (ICA, Iriarte et al., 2003).

Before sending this data to an ICA computation using the extended Infomax algorithm, bad channels were removed. The ICLabel plugin (Pion-Tonachini, Kreutz-Delgado & Makeig, 2019) was then used to test the resultant Independent Components (ICs) in order to identify and mark artefactual ICs. The bad channels that were removed prior to computing the ICA were interpolated from the remaining channels. The weights calculated during ICA were then copied to the original data. The ICs identified as artefactual were then removed from the original data. The eeguana package (Version 0.1.11.9001; Nicenboim, 2018) was then used to import the data into R (Version 4.4.3; R Core Team, 2024) for statistical analysis and epoching.

Data epochs were extracted from the continuous recordings for each participant, focusing on the critical position of the verb. These epochs spanned from 200 ms before the onset to 1200 ms after the onset (i.e., from -200 to 1200 ms). Epochs were discarded if the amplitude exceeded a threshold of 100 μV in either direction, or if the difference between the minimum and maximum amplitudes within a 200 ms window surpassed the 100 μV threshold. Additionally, trials in which the acceptability judgment task was not completed were excluded. Data from participants with too few remaining trials were also removed from further analysis. After these rejections, there were approximately 28 to 29 valid trials per condition per participant, so the number of trials included in the analysis was similar across conditions. In total, 3238 data epochs/trials were included in the analysis across participants, with a median of 807 trials per critical condition. For visualization, the valid epochs were averaged across items within each condition for each participant, and then grand averages were calculated across participants. These grand averages were smoothed with an 8 Hz low-pass filter to generate the ERP plots for the NP and verb in each condition.

ERP Data Analysis: The mean amplitudes in the time-window of interest were statistically analysed using the single trial EEG epochs at the NP and the verb for each critical condition. This was done by fitting linear mixed effects models in R (Version 4.4.3, R Core Team 2024) using the lme4 package (Bates et al., 2015). The statistical models included the fixed factors Case (Nominative vs Dative) and Verb type (Mental experiencer vs Physical experiencer), as well as the topographical factor Regions of Interest (ROI). The ROIs were defined by clustering topographically adjacent electrodes in 6 lateral and 2 midline regions. The lateral ROIs were as follows: Left-Frontal, which included electrodes E3 and E11 (which, in the 10-20 electrode system, would have been equivalent to F3 and F7); Left-Central, which included electrodes E5 and E13 (C3 and T7); Left-Parietal, which included electrodes E7 and E15 (P3 and P7); Right-Frontal, which included electrodes E4 and E12 (F4 and F8); Right-Central, which included electrodes E6 and E14 (C4 and T8); and Right-Parietal, which included electrodes E8 and E16 (P4 and P8). Mid-Fronto-Central, which included E17 and E28 (Fz and ~FCz); and Mid-Parieto-Occipital, which included E19, E20, E9, and E10 (Pz, Oz, O1 and O2), were the midline ROIs.

Instead of using a traditional subtraction-based baseline correction, we included the mean amplitude from the 200 ms pre-stimulus period (-200 to 0 ms) as a covariate (after scaling and centering) in the model for each data epoch. This approach was taken to account for and remove potential baseline differences in the statistical analysis, as suggested by Alday (2019). However, we did not interpret any effects involving pre-stimulus amplitudes, consistent with the fact that these were not part of our hypotheses. This also aligns with Alday's (2019) recommendation that "additional covariates can be included as controls without further interpretation". For the contrasts related to categorical factors, we used sum contrasts (scaled sum contrasts for 2-level factors), so that the coefficients represent deviations from the grand mean (Schad et al., 2020). Following modern statistical recommendations, we do not use the term ‘statistically significant’ or its variants for describing effects based on p-value thresholds (Wasserstein et al., 2019), but report precise p-values as continuous quantities (e.g., p = 0.06 rather than p < 0.08), unless a value is “below the limit of numerical accuracy of the data”, in which case, we report it as p < 0.001 (Amrhein et al., 2019, p.266). Further, we supplement the p-values by transforming them into s-values (Shannon information, surprisal, or binary logworth) and report s = – log_2_(p), which provides on an absolute scale, a nonprobability measure of the information provided by a p-value (Shannon, 1948; Greenland, 2019). In other words, “the s-value provides a gauge of the information supplied by a statistical test” and has the advantage of providing “a direct quantification of information without” requiring prior distributions as input (Rafi & Greenland, 2020, p.6).

**A4. Critical sentences used in the study.**

| **Dative Subject and Mental experiencer verb** | ***Nominative subject and Mental experiencer verb** |
| --- | --- |
| മാത്യുവിന് സന്തോഷം വന്നു Mathew-DAT santhosham vannu Mathew became happy. | മാത്യു സന്തോഷം വന്നു Mathew santhosham vannu Mathew became happy. |
| ബിജോയിക്ക് സന്തോഷം വന്നു Bijoy-DAT santhosham vannu. Bijoy became happy. | ബിജോയ് സന്തോഷം വന്നു Bijoy santhosham vannu. Bijoy became happy. |
| ജിനുവിന് സന്തോഷം വന്നു Jinu-Dat santhosham vannu. Jinu became happy. | ജിനു സന്തോഷം വന്നു Jinu santhosham vannu. Jinu became happy. |
| അഞ്ജലിയ്ക്ക് സന്തോഷം വന്നു Anjali-Dat santhosham vannu. Anjali became happy. | അഞ്ജലി സന്തോഷം വന്നു Anjali santhosham vannu. Anjali became happy. |
| വിമലിന് ദുഃഖം വന്നു Vimal-Dat dhukham vannu. Vimal became sad. | വിമൽ  ദുഃഖം വന്നു Vimal dhukham vannu. Vimal became sad. |
| മത്തായിക്ക് ദുഃഖം വന്നു Matthayi-Dat dhukham vannu. Matthayi became sad. | മത്തായി ദുഃഖം വന്നു Matthayi dhukham vannu. Matthayi became sad. |
| മുംതാസിന്  ദുഃഖം വന്നു Mumthas-Dat dhukham vannu Mumthas became sad. | മുംതാസ്  ദുഃഖം വന്നു Mumthas dhukham vannu Mumthas became sad. |
| അരുണിമയ്ക്ക് ദുഃഖം വന്നു Arunima-Dat dhukham vannu Arunima became sad. | അരുണിമ  ദുഃഖം വന്നു  Arunima dhukham vannu Arunima became sad. |
| ജിതിന് നാണം വന്നു Jithin-Dat nanam vannu) Jithin became shy. | ജിതിൻ  നാണം വന്നു Jithin nanam vannu. Jithin became shy. |
| സഞ്ജയ്ക്ക് നാണം വന്നു Sanjay-Dat nanam vannu. Sanjay became shy. | സഞ്ജയ് ‌  നാണം വന്നു Sanjay nanam vannu. Sanjay became shy. |
| ഷെഹനാസിന് നാണം വന്നു Shehanas -Dat nanam vannu. Shehanas became shy. | ഷഹനാസ്  നാണം വന്നു Shehanas nanam vannu. Shehanas became shy. |
| ആര്യയ്ക്ക് നാണം വന്നു Arya-Dat nanam vannu Arya became shy. | ആര്യ  നാണം വന്നു Arya nanam vannu Arya became shy. |
| ആദിലിന് വിഷമം വന്നു Adhil-Dat vishamam vannu. Adhil became sad. | ആദിൽ വിഷമം വന്നു Adhil vishamam vannu. Adhil became sad. |
| ശ്രീഹരിക്ക് വിഷമം വന്നു Sreehari-Dat vishamam vannu. Sreehari became sad. | ശ്രീഹരി വിഷമം വന്നു sreehari vishamam vannu. Sreehari became sad. |
| നൂറിന് വിഷമം വന്നു Noor-Dat vishamam vannu Noor became sad. | നൂർ  വിഷമം വന്നു Noor vishamam vannu Noor became sad. |
| സൗദയ്ക്ക് വിഷമം വന്നു Soudha-Dat vishamam vannu Soudha became sad. | സൗദ  വിഷമം വന്നു Soudha vishamam vannu Soudha became sad. |
| റോബിന് അത്ഭുതം വന്നു Robin-Dat athbhutham vannu. Robin became surprised. | റോബിൻ അത്ഭുതം വന്നു Robin athbhutham vannu. Robin became surprised. |
| ആന്റണിക്ക് അത്ഭുതം വന്നു Antony-Dat athbhutham vannu. Antony became surprised. | ആന്റണി അത്ഭുതം വന്നു Antony athbhutham vannu. Antony became surprised. |
| നസ്രിന് അത്ഭുതം വന്നു Nazrin-Dat athbhutham vannu Nazrin became surprized. | നസ്രിൻ അത്ഭുതം വന്നു Nazrin athbhutham vannu Nazrin became surprized. |
| ശ്രീകലയ്ക്ക് അത്ഭുതം വന്നു Sreekala-Dat athbhutham vannu Sreekala became surprized. | ശ്രീകല അത്ഭുതം വന്നു Sreekala athbhutham vannu Sreekala became surprized. |
| ആസിഫിന് കൊതി വന്നു Asif-Dat kothi vannu.  Asif felt a desire | ആസിഫ്  കൊതി വന്നു Asif kothi vannu.  Asif felt a desire |
| ബിനോയ്‌ക്ക്‌ കൊതി വന്നു Binoy-Dat kothi vannu.  Binoy felt a desire | ബിനോയ് ‌ കൊതി വന്നു Binoy kothi vannu.  Binoy felt a desire. |
| ബിക്കുവിന് കൊതി വന്നു Bikku-Dat kothi vannu.  Bikku felt a desire | ബിക്കു  കൊതി വന്നു Bikku kothi vannu.  Bikku felt a desire |
| അനുപമയ്‌ക്ക് കൊതി വന്നു Anupama-Dat kothi vannu.  Anupama felt a desire | അനുപമ കൊതി വന്നു Anupama kothi vannu.  Anupama felt a desire |
| അനന്ദുവിന്‌ വിരഹം വന്നു Anandhu-Dat viraham vannu  Anandhu felt longing. | അനന്ദു ‌ വിരഹം വന്നു Anandhu viraham vannu  Anandhu felt longing. |
| ടോവിനോയ്ക്ക് വിരഹം വന്നു Tovino-Dat viraham vannu.  Tovino felt longing. | ടോവിനോ വിരഹം വന്നു Tovino viraham vannu.  Tovino felt longing |
| ടിന്റുവിന് വിരഹം വന്നു Tintu-Dat viraham vannu.  Tintu felt longing. | ടിന്റു വിരഹം വന്നു Tintu viraham vannu.  Tintu felt longing |
| സർഗയ്ക്ക് വിരഹം വന്നു Sarga-Dat viraham vannu.  Sarga felt longing. | സർഗ്ഗ  വിരഹം വന്നു Sarga viraham vannu.  Sarga felt longing. |
| മഹേഷിന് ആശ്വാസം വന്നു Mahesh-Dat aashwasam vannu.  Mahesh felt relief. | മഹേഷ് ആശ്വാസം വന്നു Mahesh-Dat aashwasam vannu.  Mahes felt relief. |
| വിനോയ്ക്ക് ആശ്വാസം വന്നു Vinoy-Dat aashwasam vannu.  Vinoy felt relief | വിനോയ് ആശ്വാസം വന്നു Vinoy aashwasam vannu.  Vinoy felt relief |
| നീതുവിന് ആശ്വാസം വന്നു Neethu-Dat aashwasam vannu.  Neethu felt relief | നീതു  ആശ്വാസം വന്നു Neethu aashwasam vannu  Neethu felt relief |
| അനഘയ്ക്ക് ആശ്വാസം വന്നു Anagha-Dat ashwasam vannu.  Anagha felt relief | അനഘ ആശ്വാസം വന്നു Anagha ashwasam vannu.  Anagha felt relief |
| ഹരീഷിന് നിരാശ വന്നു Hareesh-Dat nirasha vannu.  Harish was disappointed. | ഹരീഷ് നിരാശ വന്നു Hareesh nirasha vannu.  Harish was disappointed. |
| ബ്ലെസ്സ്ലിയ്ക്ക് നിരാശ വന്നു Blessly-Dat nirasha vannu.  Blessly was disappointed. | ബ്ലെസ്സ്ലി നിരാശ വന്നു Blessly nirasha vannu.  Blessly was disappointed. |
| സീതുവിന്‌ നിരാശ വന്നു Seethe-Dat nirasha vannu.  Seethu was disappointed. | സീതു ‌ നിരാശ വന്നു Seethu nirasha vannu.  Seethu was disappointed. |
| അമലയ്ക്ക് നിരാശ വന്നു Amala-Dat nirasha vannu.  Amala was disappointed. | അമല നിരാശ വന്നു Amala-Dat nirasha vannu.  Amala was disappointed. |

| **Dative Subject and Physical experiencer verb** | ***Nominative subject and physical experiencer verb** |
| --- | --- |
| മാത്യുവിന് പനി വന്നു Mathew-DAT pani vannu Mathew became feverish | മാത്യു പനി വന്നു Mathew pani vannu Mathew became feverish. |
| ബിജോയിക്ക്  പനി വന്നു Bijoy-DAT pani vannu. Bijoy became feverish | ബിജോയ് പനി വന്നു Bijoy pani vannu. Bijoy became feverish. |
| ജിനുവിന്  പനി വന്നു Jinu-Dat pani vannu. Jinu became feverish. | ജിനു പനി വന്നു Jinu pani vannu. Jinu became feverish. |
| അഞ്ജലിയ്ക്ക്  പനി വന്നു Anjali-Dat pani vannu. Anjali became feverish. | അഞ്ജലി പനി വന്നു Anjali pani vannu. Anjali became feverish. |
| വിമലിന് വിശപ്പ് വന്നു Vimal-Dat vishappu vannu. Vimal became hungry. | വിമൽ  വിശപ്പ് വന്നു Vimal vishappu vannu. Vimal became hungry. |
| മത്തായിക്ക്  വിശപ്പ് വന്നു Matthayi-Dat vishappu vannu. Matthayi became hungry | മത്തായി  വിശപ്പ് വന്നു Matthayi visahppu vannu. Matthayi became hunry. |
| മുംതാസിന് വിശപ്പ് വന്നു Mumthas-Dat vishappu vannu Mumthas became hungry. | മുംതാസ്  വിശപ്പ് വന്നു Mumthas visahppu vannu Mumthas became hungry. |
| അരുണിമയ്ക്ക് വിശപ്പ് വന്നു Arunima-Dat vishappu vannu Arunima became hungry. | അരുണിമ  വിശപ്പ് വന്നു Arunima vishappu vannu Arunima became hungry. |
| ജിതിന് ദാഹം വന്നു Jithin-Dat dhaham vannu Jithin became thirsty. | ജിതിൻ  ദാഹം വന്നു Jithin dhaham vannu. Jithin became thirsty |
| സഞ്ജയ്ക്ക് ദാഹം വന്നു Sanjay-Dat dhaham vannu. Sanjay became thirsty. | സഞ്ജയ് ‌  ദാഹം വന്നു Sanjay dhaham vannu. Sanjay became thirsty. |
| ഷെഹനാസിന് ദാഹം വന്നു Shehanas -Dat dhaham vannu. Shehanas became thirsty. | ഷഹനാസ്  ദാഹം വന്നു Shehanas dhaham vannu. Shehanas became thirsty. |
| ആര്യയ്ക്ക് ദാഹം വന്നു Arya-Dat dhaham vannu Arya became thirsty. | ആര്യ  ദാഹം വന്നു Arya dhaham vannu Arya became thirsty. |
| ആദിലിന് കിതപ്പ് വന്നു Adhil-Dat kithappu vannu. Adhil became dizzy | ആദിൽ  കിതപ്പ് വന്നു Adhil kitham vannu. Adhil became dizzy. |
| ശ്രീഹരിക്ക് കിതപ്പ് വന്നു Sreehari-Dat kithappu vannu. Sreehari became dizzy. | ശ്രീഹരി കിതപ്പ് വന്നു sreehari kithappu vannu. Sreehari became dizzy. |
| നൂറിന് കിതപ്പ് വന്നു Noor-Dat kithappu vannu Noor became dizzy. | നൂർ  കിതപ്പ് വന്നു Noor kithappu vannu Noor became dizzy. |
| സൗദയ്ക്ക് കിതപ്പ് വന്നു Soudha-Dat kithappu vannu Soudha became dizzy | സൗദ  കിതപ്പ് വന്നു Soudha kithappu vannu Soudha became dizzy. |
| റോബിന് തളർച്ച വന്നു Robin-Dat thalarcha vannu. Robin became tired | റോബിൻ തളർച്ച വന്നു Robin thalarcha vannu. Robin became tired |
| ആന്റണിക്ക് തളർച്ച വന്നു Antony-Dat thalarcha vannu. Antony became tired | ആന്റണി തളർച്ച വന്നു Antony thalarcha vannu. Antony became tired. |
| നസ്രിന് തളർച്ച വന്നു Nazrin-Dat thalarcha vannu Nazrin became tired. | നസ്രിൻ തളർച്ച വന്നു Nazrin thalrcha vannu Nazrin became tired |
| ശ്രീകലയ്ക്ക് തളർച്ച വന്നു Sreekala-Dat thalarcha vannu Sreekala became tired. | ശ്രീകല തളർച്ച വന്നു Sreekala thalrcha vannu Sreekala became tired |
| ആസിഫിന് വിറയൽ വന്നു Asif-Dat virayal vannu.  Asif felt shivering | ആസിഫ്  വിറയൽ വന്നു Asif virayal vannu.  Asif felt shivering |
| ബിനോയ്‌ക്ക്‌ വിറയൽ വന്നു Binoy-Dat virayal vannu.  Binoy felt shivering. | ബിനോയ് ‌ വിറയൽ വന്നു  Binoy virayal vannu  Binoy Binoy felt shivering. |
| ബിക്കുവിന് വിറയൽ വന്നു  Bikku-Dat virayal vannu.  Bikku felt shivering. | ബിക്കു  വിറയൽ വന്നു Bikku virayal vannu.  Bikku felt shivering. |
| അനുപമയ്‌ക്ക് വിറയൽ വന്നു Anupama-Dat virayal vannu.  Anupama felt shivering. | അനുപമ വിറയൽ വന്നു Anupama virayal vannu.  Anupama felt shivering. |
| അനന്ദുവിന്‌ കുളിര് വന്നു Anandhu-Dat kuliru vannu  Anandhu felt cold. | അനന്ദു ‌  കുളിര് വന്നു Anandhu kuliru vannu  Anandhu felt cold. |
| ടോവിനോയ്ക്ക് കുളിര് വന്നു Tovino-Dat kuliru vannu.  Tovino felt cold. | ടോവിനോ കുളിര് വന്നു Tovino kuliru vannu.  Tovino felt cold. |
| ടിന്റുവിന് കുളിര് വന്നു Tintu-Dat kuliru vannu.  Tintu felt cold. | ടിന്റു കുളിര് വന്നു Tintu kuliru vannu.  Tintu felt cold. |
| സർഗയ്ക്ക് കുളിര് വന്നു Sarga-Dat kuliru vannu.  Sarga felt cold. | സർഗ്ഗ  കുളിര് വന്നു Sarga kuliru vannu.  Sarga felt cold. |
| മഹേഷിന് തലവേദന വന്നു  Mahesh-Dat thalavedhana vannu.  Mahesh felt a headache. | മഹേഷ് തലവേദന വന്നു Mahesh-Dat thalavedhana vannu.  Mahesh felt a headache. |
| വിനോയ്ക്ക് തലവേദന വന്നു Vinoy-Dat thalavedhana vannu.  Vinoy felt a headache. | വിനോയ് തലവേദന വന്നു Vinoy thalavedhana vannu.  Vinoy felt a headache. |
| നീതുവിന് തലവേദന വന്നു Neethu-Dat thalavedhana vannu.  Neethu felt a headache. | നീതു  തലവേദന വന്നു Neethu thalavedhana vannu  Neethu felt a headache. |
| അനഘയ്ക്ക് തലവേദന വന്നു  Anagha-Dat thalavedhana vannu.  Anagha felt headache. | അനഘ തലവേദന വന്നു  Anagha thalavedhana vannu.  Anagha felt headache. |
| ഹരീഷിന് വിളർച്ച വന്നു Hareesh-Dat vilarcha vannu.  Hareesh became anaemic. | ഹരീഷ് വിളർച്ച വന്നു  Hareesh vilarcha vannu.  Hareesh became anemic |
| ബ്ലെസ്സ്ലിയ്ക്ക് വിളർച്ച വന്നു Blessly-Dat vilarcha vannu.  Blessly became anaemic. | ബ്ലെസ്സ്ലി വിളർച്ച വന്നു Blessly vilarcha vannu.  Blessly became anaemic. |
| സീതുവിന്‌ വിളർച്ച വന്നു Seethu-Dat viarcha vannu.  Seethe became anaemic | സീതു ‌ വിളർച്ച വന്നു Seethu vilarcha vannu.  Seethe became anaemic |
| അമലയ്ക്ക് വിളർച്ച വന്നു Amala-Dat vilarcha vannu.  Amala became anaemic | അമല വിളർച്ച വന്നു Amala-Dat vilarcha vannu.  Amala became anaemic. |

**A5. ERPs at the sentence-initial subject noun**

Figure. S1 shows the ERPs at the position of the sentence-initial subject noun. We computed a linear mixed effects model with fixed factors Case of the subject noun, ROI, the -200-0 ms pre-stimulus baseline mean amplitude as a covariate (scaled and centred), and by-participant and by-item random intercepts. The analysis code and full model outputs are available as R notebooks in the analysis repository online. Type II Wald Chi-squared tests on this model (AIC = 399983.75) showed an interaction effect of ROI x Case (χ^2^(1) = 35.56, p < 0.001, s = 16.80). Estimated marginal means on the response scale were computed on the model using the emmeans package (Lenth, 2021) to resolve this interaction. The pairwise contrasts of estimates for Case within each level of ROI revealed simple effects of Case in the left-frontal region (estimate = -0.817, SE = 0.326, p = 0.01, s = 6.35), right-parietal region (estimate = 0.613, SE = 0.326, p = 0.06, s = 4.05) and mid-fronto-central (estimate = -0.731, SE = 0.326, p = 0.02, s = 5.32) region. The estimate for the dative subjects were more negative than those for the nominative subjects in the left-frontal and mid-fronto-central region. In the right-parietal region, the estimate for dative subjects is less positive than that for the nominative subjects. As the effects at the sentence-initial noun do not form part of our original hypotheses at the verb position, we refrain from interpreting them further. A more complex linear mixed-effects model was computed, including by-participant random slopes for Case^[[2]](#footnote-2)^, which showed that this pattern of results remained largely intact despite numerical differences.

**
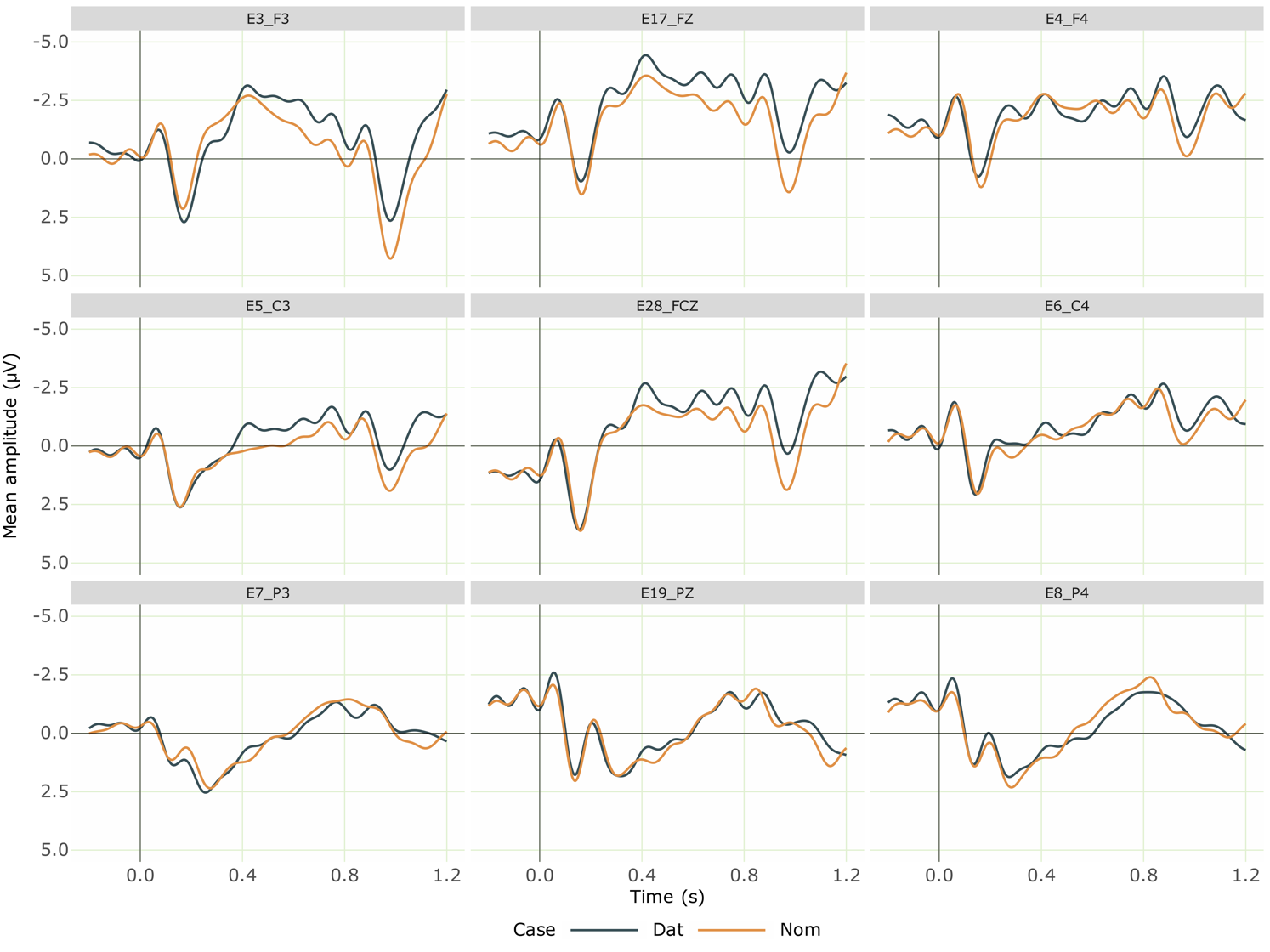
**

**Figure S1. Grand averaged ERPs at the nominative and dative sentence-initial subject nouns from 28 participants. Negativity is plotted upwards; the time axis runs from -0.2 s to 1.2 s (i.e., -200 ms to 1200 ms) with 0 being the onset of the noun. The dark blue line shows the ERPs for the dative nouns and the orange line shows that for the nominative nouns.**

**A6. Absence of late positivity effects at the Verb**

Figure. S2 and Figure. S3 show the ERP at the verb for mental and physical experiencer verb conditions separately respectively. The study revealed no late positivity effects, which is a similar finding to that reported by Shalu et al. (2025). We observed only an N400 despite the understanding that the ill-formed sentences typically elicit a P600 effect (Bornkessel-Schlesewsky & Schlesewsky, 2008). The absence of P600 effects can be potentially explained by the resolvability of the violation condition (Frenzel et al., 2011; Bornkessel et al., 2011). The violation constructions in the experiment can be non-anomalous when we use them in a different context such as a serial verb construction, as in (A-C).

1. രാമൻ സന്തോഷം വന്നു തുള്ളി ചാടി.

Raman santhosham vannu thulli chadi.

Raman(an.m.nom)happiness come-frozen- pst jump-Frozen- pst Jump- pst

Raman jumped with joy.

1. രാമൻ വിശപ്പ് വന്നു തളർന്നു കിടന്നു

Raman vishappu vannu thalrnnu kidannu.

Raman(an.m.nom) hunger come-Frozen-pst faint-Frozen-pst lie-Frozen-pst

Raman became hungry and collapsed from exhaustion.

1. രാമൻ വിശപ്പ് വന്നു പത്തിരിയും ഇറച്ചിയും കഴിച്ചു.

Raman vishappu vannu pathiriyum erachiyum kazhichu.

Raman(an.m.nom) hunger come.PST bread-conj meat-conj eat.PST

“ Raman got hungry and ate bread and meat”.

**ERPs at the verb: Mental experiencer verbs**

**
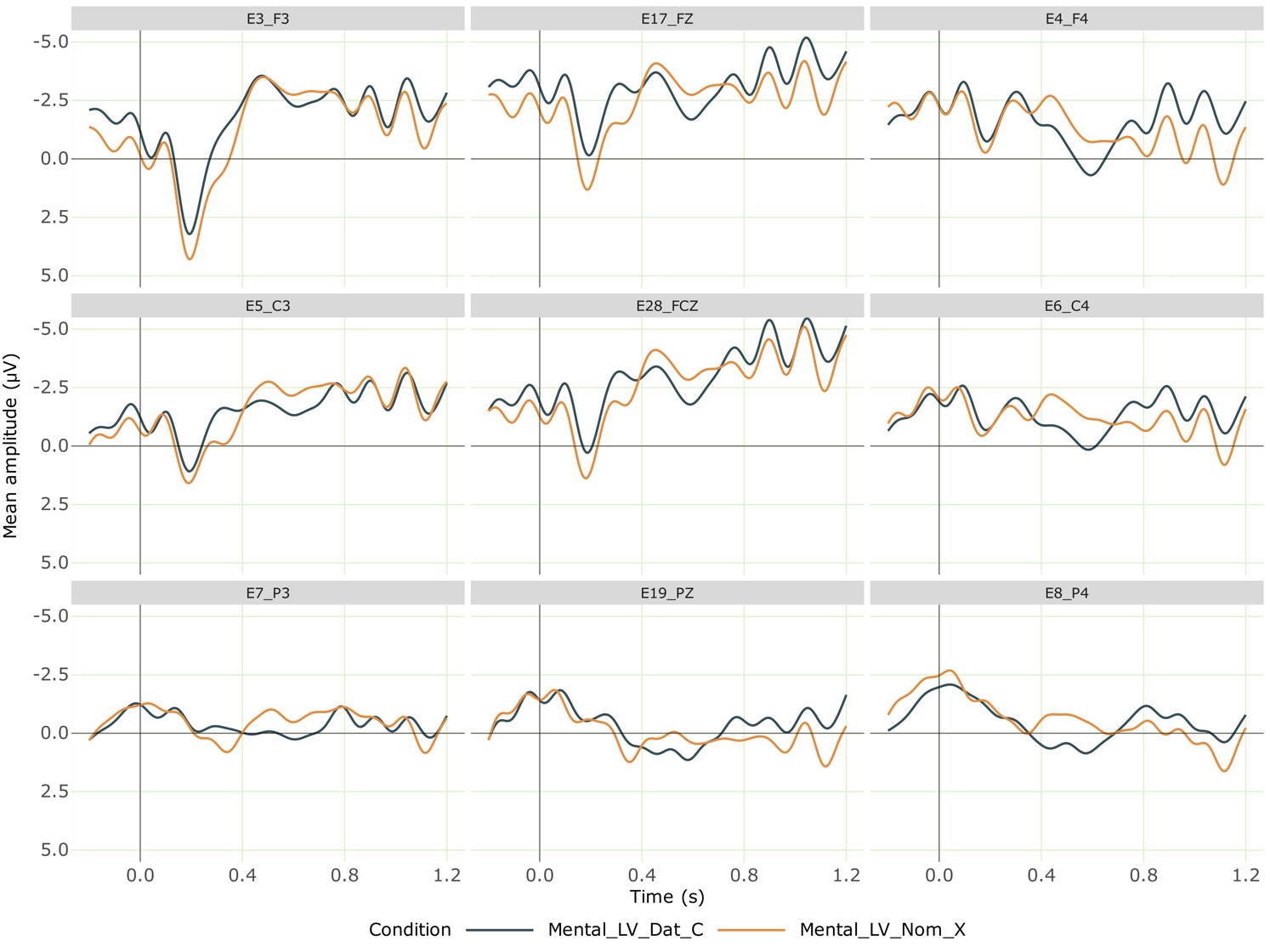
**

**Figure S2. Grand averaged ERPs at the position of Mental experiencer light verb comparing Dative mental experiencer light verb (Correct) and Nominative mental experiencer light verb (Incorrect) from 28 participants. Negativity is plotted upwards; the time axis runs from -0.2 s to 1.2 s (i.e., -200 ms to 1200 ms) with 0 being the onset of the critical verb. The dark blue line shows the ERPs for the correct Mental experiencer verb (with a dative subject) and the orange line shows that for incorrect mental experiencer verb (with a nominative subject), which elicited a negativity effect.**

**ERPs at the verb: Physical experiencer verbs**


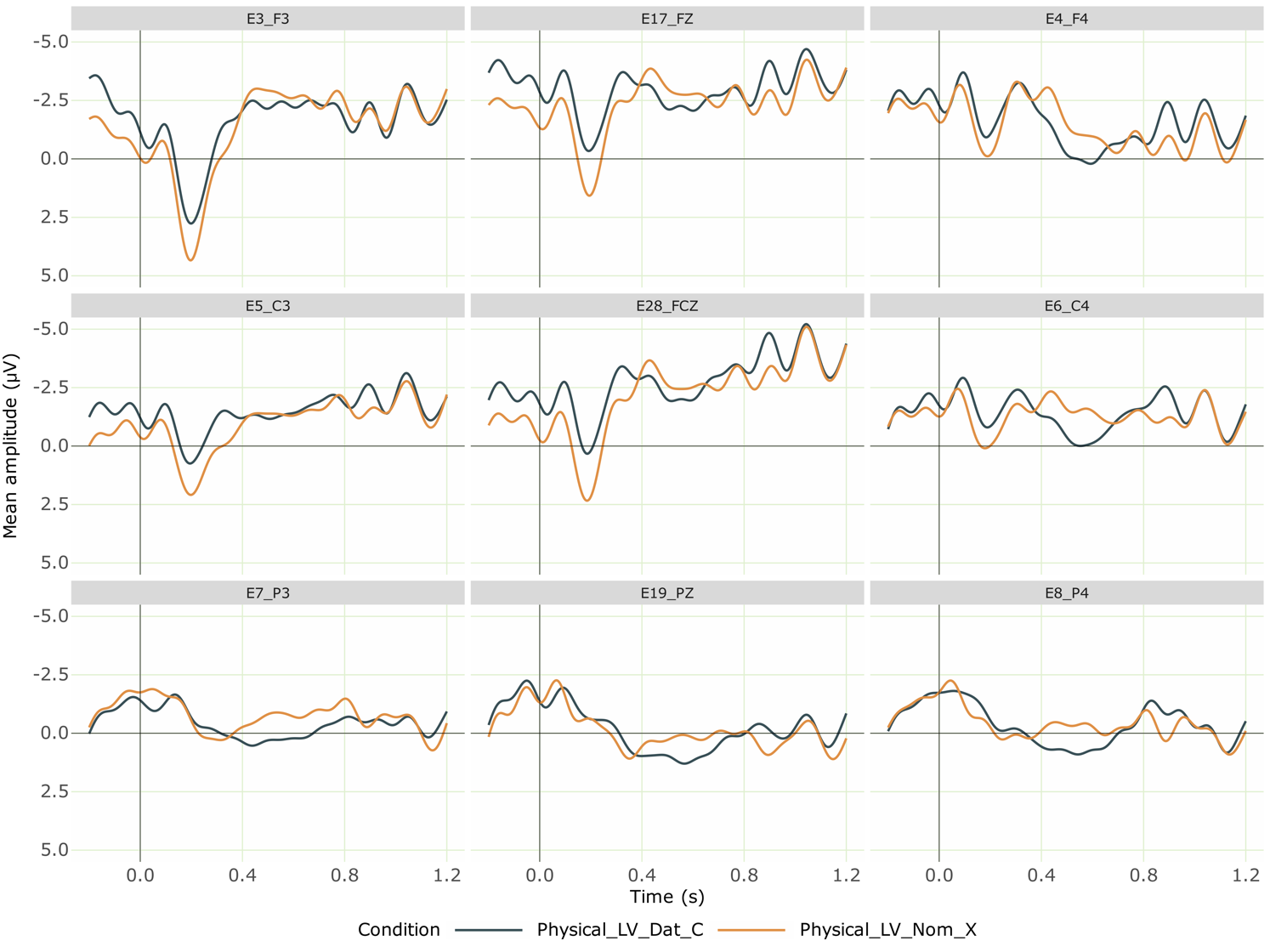


**Figure S3. Grand averaged ERPs at the position of Physical experiencer light verb comparing Dative Physical experiencer light verb (Correct) and Nominative Physical experiencer light verb (Incorrect) from 28 participants. Negativity is plotted upwards; the time axis runs from -0.2 s to 1.2 s (i.e., -200 ms to 1200 ms) with 0 being the onset of the critical verb. The dark blue line shows the ERPs for the correct Physical experiencer light verb (with a dative subject) and the orange line shows that for incorrect Physical experiencer light verb (with a nominative subject), which elicited a negativity.**


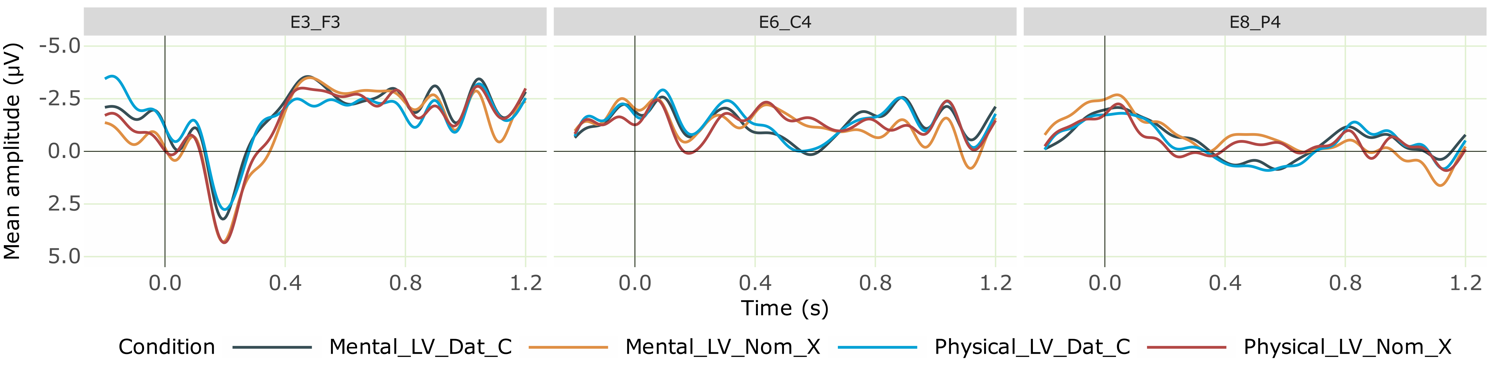
**ERPs at the verb: All conditions**

**Figure S4**. **Grand averaged ERPs at the verb for the critical conditions from 28** **participants**. **Negativity is plotted upwards; the time axis runs from -0.2 s to** 1.2 **s (i.e., -200 ms to 1200 ms) with 0 being the onset of the critical verb. The** **dark blue line represents the correct mental experiencer light verb (with a dative subject), while the orange line shows the incorrect mental experiencer light verb (with a nominative subject), which elicited a centro-parietal negativity effect. The light blue line indicates the correct physical experiencer verb (with a dative subject) and the red line represents the incorrect physical experiencer verb (with a nominative subject), which elicited a centro-parietal negativity effect. Regardless of grammaticality, conditions with mental experiencer light verbs elicited a further negativity effect in the left anterior region compared to physical experiencer light verbs.**

**References**

Oldfield, R. C. (1971). Edinburgh handedness inventory. *Journal of Abnormal Psychology*.

Rajendran, S. (1977). Case System in Tamil and Malayalam. *Bulletin of the Deccan College Research Institute*, *37*(1/4), 128-140.

Muralikrishnan, R., & Idrissi, A. (2021). Salience-weighted agreement feature hierarchy modulates language comprehension. In Cortex (Vol. 141, pp. 168–189). Elsevier BV. <https://doi.org/10.1016/j.cortex.2021.03.029>

Demiral, Ş. B., Schlesewsky, M., & Bornkessel-Schlesewsky, I. (2008). On the universality of language comprehension strategies: Evidence from Turkish. In Cognition (Vol. 106, Issue 1, pp. 484–500). Elsevier BV. <https://doi.org/10.1016/j.cognition.2007.01.008>

Bornkessel-Schlesewsky, I., Roehm, D., Mailhammer, R., & Schlesewsky, M. (2020). Language Processing as a Precursor to Language Change: Evidence From Icelandic. In Frontiers in Psychology (Vol. 10). Frontiers Media SA. <https://doi.org/10.3389/fpsyg.2019.03013>

Ferree, T. C., Luu, P., Russell, G. S., & Tucker, D. M. (2001). Scalp electrode impedance, infection risk, and EEG data quality. *Clinical neurophysiology*, *112*(3), 536-544.

Delorme, A., & Makeig, S. (2004). EEGLAB: an open source toolbox for analysis of single-trial EEG dynamics including independent component analysis. *Journal of neuroscience methods*, *134*(1), 9-21.

Delorme, A. (2023). EEG is better left alone. In Scientific Reports (Vol. 13, Issue 1). Springer Science and Business Media LLC. <https://doi.org/10.1038/s41598-023-27528-0>

Roehm, D., Winkler, T., Swaab, T., & Klimesch, W. (2002). The N400 and delta oscillations: Is there a difference?. *Journal of Cognitive Neuroscience*, 134-135.

Iriarte, J., Urrestarazu, E., Valencia, M., Alegre, M., Malanda, A., Viteri, C., & Artieda, J. (2003). Independent component analysis as a tool to eliminate artifacts in EEG: a quantitative study. *Journal of clinical neurophysiology*, *20*(4), 249-257.

Pion-Tonachini, L., Kreutz-Delgado, K., & Makeig, S. (2019). ICLabel: An automated electroencephalographic independent component classifier, dataset, and website. *NeuroImage*, *198*, 181-197.

Nicenboim, B. (2018). eeguana: A package for manipulating EEG data in R. *Computer software* Version 0.1.11.9001. *Retrieved from https://github. com/bnicenboim/eeguana*. <http://doi.org/10.5281/zenodo.2533138>

R Core Team (2024). R: A Language and Environment for Statistical Computing. R Foundation for Statistical Computing. <https://www.R-project.org/>

Bates, D., Mächler, M., Bolker, B., & Walker, S. (2015). Fitting Linear Mixed-Effects Models Using lme4. In Journal of Statistical Software (Vol. 67, Issue 1). Foundation for Open Access Statistic. <https://doi.org/10.18637/jss.v067.i01>

Alday, P. M. (2019). How much baseline correction do we need in ERP research? Extended GLM model can replace baseline correction while lifting its limits. In Psychophysiology (Vol. 56, Issue 12). Wiley. <https://doi.org/10.1111/psyp.13451>

Schad, D. J., Vasishth, S., Hohenstein, S., & Kliegl, R. (2020). How to capitalize on a priori contrasts in linear (mixed) models: A tutorial. In Journal of Memory and Language (Vol. 110, p. 104038). Elsevier BV. <https://doi.org/10.1016/j.jml.2019.104038>

Amrhein, V., Trafimow, D., & Greenland, S. (2019). Inferential Statistics as Descriptive Statistics: There Is No Replication Crisis if We Don’t Expect Replication. In The American Statistician (Vol. 73, Issue sup1, pp. 262–270). Informa UK Limited. <https://doi.org/10.1080/00031305.2018.1543137>

Shannon, C. E. (1948). A mathematical theory of communication. *The Bell system technical journal*, *27*(3), 379-423.

Greenland, S. (2019). Valid p-values behave exactly as they should: Some misleading criticisms of p-values and their resolution with s-values. *The American Statistician*, *73*(sup1), 106-114.

Rafi, Z., & Greenland, S. (2020). Semantic and cognitive tools to aid statistical science: replace confidence and significance by compatibility and surprise. In BMC Medical Research Methodology (Vol. 20, Issue 1). Springer Science and Business Media LLC. <https://doi.org/10.1186/s12874-020-01105-9>

Lenth Russell, V. (2021). emmeans: Estimated Marginal Means, aka Least-Squares Means. R package version 1.6. 0.

Shalu, S., Muralikrishnan, R., Mathew, A. M., & Choudhary, K. K. (2025). Similar but different: ERP evidence on the processing of mental and physical experiencer verbs in Malayalam. Preprint of submitted article. bioarxiv.org. Cold Spring Harbor Laboratory. https://doi.org/10.1101/2025.03.12.642939

Bornkessel-Schlesewsky, I., & Schlesewsky, M. (2008). An alternative perspective on “semantic P600” effects in language comprehension. *Brain research reviews*, *59*(1), 55-73.

Frenzel, S., Schlesewsky, M., & Bornkessel-Schlesewsky, I. (2011). Conflicts in language processing: A new perspective on the N400–P600 distinction. *Neuropsychologia*, *49*(3), 574-579.

Bornkessel-Schlesewsky, I., Kretzschmar, F., Tune, S., Wang, L., Genç, S., Philipp, M., ... & Schlesewsky, M. (2011). Think globally: Cross-linguistic variation in electrophysiological activity during sentence comprehension. *Brain and language*, *117*(3), 133-152.

1. The usage of these case suffixes is purely based on the phonology (last syllable) of the noun to which these are attached, regardless of the gender, number and other properties of the noun concerned. The allomorph “ə” is used for nouns or their derivation ending in “n”, while “-kkə” is applied elsewhere (Rajendran,1977). [↑](#footnote-ref-1)
2. The full model output for the complex model is provided for reference in the analysis repository online. [↑](#footnote-ref-2)
